# Understanding middle managers’ influence in implementing patient safety culture

**DOI:** 10.1186/s12913-017-2533-4

**Published:** 2017-08-22

**Authors:** Jennifer Gutberg, Whitney Berta

**Affiliations:** Institute of Health Policy, Management and Evaluation, Dalla Lana School of Public Health, University of Toronto, 155 College Street, Toronto, ON M5T 3M6 Canada

## Abstract

**Background:**

The past fifteen years have been marked by large-scale change efforts undertaken by healthcare organizations to improve patient safety and patient-centered care. Despite substantial investment of effort and resources, many of these large-scale or “radical change” initiatives, like those in other industries, have enjoyed limited success – with practice and behavioural changes neither fully adopted nor ultimately sustained – which has in large part been ascribed to inadequate implementation efforts. Culture change to “patient safety culture” (PSC) is among these radical change initiatives, where results to date have been mixed at best.

**Discussion:**

This paper responds to calls for research that focus on explicating factors that affect efforts to implement radical change in healthcare contexts, and focuses on PSC as the radical change implementation. Specifically, this paper offers a novel conceptual model based on Organizational Learning Theory to explain the ability of middle managers in healthcare organizations to influence patient safety culture change.

**Summary:**

We propose that middle managers can capitalize on their unique position between upper and lower levels in the organization and engage in ‘ambidextrous’ learning that is critical to implementing and sustaining radical change. This organizational learning perspective offers an innovative way of framing the mid-level managers’ role, through both explorative and exploitative activities, which further considers the necessary organizational context in which they operate.

## Background

Leadership is one of the most seemingly impactful and frequently cited factors associated with large-scale, ‘radical’ organizational change [1, 2]. The attention given to leadership in implementation and change management research suggests that it plays a vital role in affecting significant reforms in organizations, notably in organizational culture change efforts [3–11]. Theories of both implementation and change management further support the purported importance of leadership at all levels facilitating such radical change, in particular leadership at the level of middle management – those with supervisory or managerial roles that are neither front-line workers nor senior leadership team members [12, 13]. A recent body of evidence in healthcare suggests that engagement of leadership at multiple levels of an organization is critical in affecting the success of strategic initiatives aimed at large-scale change, and in sustaining these changes long-term [14].

In spite of the increasing knowledge around the value of middle management both within and beyond the healthcare sector, our understanding of middle managers’ contributions to strategy and organizational change remains limited [12, 13, 15, 16], despite numerous calls in the popular/lay literature to engage middle managers as a vital mechanism for effecting strategic change [17]. The need for senior leadership and a strong vision in creating change has been well established, yet the persistent challenge of sustaining large-scale change initiatives [18–20] does underscore the importance of considering whether senior leadership may be a “necessary but insufficient” condition to enact meaningful sustained change. Further, given the increased focus in healthcare on team- and clinical microsystem-level functioning, leadership is becoming even more diffuse across the organization [21] and by necessity middle management will play an increasingly significant role in improving organizations.

In the context of healthcare organizations, middle managers often have to work within highly hierarchical structures that are generally siloed by professional group (e.g., doctors, nurses, allied health professionals). In addition to having to balance the sometimes competing interests of these groups – and in fact often themselves being members of specialized professional groups, whose chief roles relate to patient care and not the conduct of strategic activities [22] – middle managers also face unique contextual constraints. That is, though middle managers have been described as having a ‘semi-autonomous’ role [23], in healthcare in particular middle managers are beholden to their profession, their patients, and senior management – exemplifying a phenomenon described by some organization scientists as “stuck in the middle” [24].

These contrasting views regarding middle managers’ potential strategic value to organizations, against the unique contextual challenges they face, beg the question: how can middle managers in healthcare organizations influence organizational change, and under what circumstances will they be most effective in affecting this change?

In order to address this question in a practical and relevant context, this paper presents the issue of organizational change relating specifically to patient safety, focusing on discussion of middle managers roles in the implementation of strategies aimed at creating and improving patient safety culture. This focus, we think, is timely: as explained by Benn et al., “there is now growing recognition that patient safety and the capacity of an organisation to deliver consistent, high-quality and failure-free care is both a systemic issue and one that needs to be addressed at the level of the whole organisation or care system” [25] (p.1767).

The paper will begin by framing the context of patient safety efforts and extant literature that reviews these efforts, followed by a review of the relevant evidence on middle management’s involvement in change initiatives in healthcare. Together, these background elements will be used to justify the proposition of a theoretical framework for middle managers’ contributions to organizational change, predicated on Organizational Learning Theory [26]. Organizational learning theory has been widely used across multiple sectors to explore large-scale change [26, 27], and emphasizes the need for organizations to explore both radical/whole system, and incremental, change innovations [26, 28, 29].

## What we know about patient safety change initiatives

In 1999, the Institute of Medicine released its seminal publication on patient safety and adverse events along with subsequent reports on healthcare quality [30–32]. Since those reports first emerged, these issues have become top of mind for almost every healthcare organization, including acute care facilities. The reports and others like them that followed [33] focused on elements for total redesign or ‘radical change’ of the system that put patient safety at the forefront of healthcare, and advocated changing from a culture of blame and fear to one of accountability, safety, and patient-centeredness [30]. As a result, patient safety has become ubiquitous and with it the effort to build patient safety cultures (PSC) [31, 34, 35].

Despite over 15 years of progress, significant issues still exist with the conceptualization and execution of PSC initiatives and translating these to front-line outcomes. While there have been numerous successful efforts to change organizations’ cultures, the current view remains that these efforts are often not fully sustainable and changes are not being adopted effectively [36, 37]. In fact, some reports have found that while patient safety awareness has increased, implementation effectiveness and patient and organizational performance outcomes have actually worsened in areas [18, 38]. It is clear that effecting PSC change is a challenging undertaking, and that our understanding of the factors that affect efforts to adopt PSC is limited.

The challenges in both measuring and improving organizational culture (and in turn PSC) are well documented within and beyond the healthcare literature [39, 40]. The fundamental challenge results first from methodological difficulty in operationalizing organizational culture [41], as well as empirically validating a cause-and-effect relationship [25, 42]. Mearns and Flin suggest that safety culture in particular has its own set of methodological challenges, where efforts to assess a “safety culture” are often met with measurement of safety attitudes, with little attention paid to the norms, values, and rules that in fact embody a culture [41].

Though these perspectives are critical in any empirical or theoretical examination of organizational culture, a body of evidence does exist to support a) empirical efforts to improve organizational culture generally and patient safety culture specifically, and b) targeted strategic efforts aimed at improving culture [40, 43]. In particular, this has been manifested in much of the existing literature as concrete strategies such as unit-level programs, team-based interventions [44, 45], and broader organizational interventions (which could include single or multi-component interventions [40]) [43]**.** Though as mentioned above, the success of these initiatives is variable at best, this paper suggests that this is a result of implementation challenges – and specifically the relative absence of consideration of middle managers in effecting culture change – rather than a reflection of the interventions themselves. In light of this, the following section will offer an overview of the evidence around middle management’s potential and realized contribution to organizational change, culture change, and strategy implementation.

Leadership’s significance to broader organizational change in healthcare has previously been discussed herein. However, this link between leadership and patient safety culture change efforts in particular has also been examined. One systematic review found that leadership, in the form of executive walkrounds, was one of the strategies used to improve patient safety culture, though the evidence only found partial support for change in PSC climate [40]. Weaver et al. conducted another systematic review with similar aims, which found similar evidence for the effectiveness of leadership interventions – again in the form of executive walkrounds – in the improvement of perceptions of safety culture, as well as patient outcomes [43]. It appears that interventions to improve patient safety culture focused on leadership seem to focus on the executive level. However, the potential value of non-senior leaders in influencing patient safety culture has also been considered: Kaplan et al. explain leadership drives patient safety culture, but add that “…one must look beyond the CEO and consider the roles of the entire executive team, the trustees or directors, and the leaders who are charged with managing teams in the organization. Strong leadership is also shared leadership…” In order to explore how these non-senior leaders (in particular middle management) may contribute to patient safety culture change efforts, we review the extant literature on middle management’s role in change in healthcare.

## Middle management’s role in change initiatives in healthcare

In order to understand middle management’s potential contribution to patient safety culture efforts, the population being explored must first be defined. There is a significant gap in the literature regarding the identification of middle managers, which is explained through the following quote by McConville:

“Middle managers are more difficult to distinguish, as the boundaries between levels of hierarchy are often blurred. The exercise is further complicated in organisations with organic structures where demarcation may be ambiguous. As a result few writers have attempted to define the role. Middle line management is often described in terms of what it is not” [46] (p.639).

Indeed, a review of the literature finds that studies of “middle managers” in healthcare include undefined middle managers [47–51]; nurse managers [52–54]; ‘unit-level’ managers [55–57]; ward managers [23]; department leads [58]; clinical directors [59, 60]; facility manager [61]; as well as idiosyncratically-defined middle managers (i.e., where the authors intentionally set bounded criteria, possibly guided by the organization(s) in question being studies [16, 46]). Though each level of management described above has a bounded set of responsibilities and may be more or less involved in regular strategic activities of the organization, the literature linking middle managers to organizational change does not appear to set limits on which middle managers would be able to contribute to organizational culture change. As a result, we consider “middle managers” herein as any individual in an organization who operates above the front-line of the organization, but below the level of senior management.

The potential value for middle managers to contribute to strategic organizational activities in healthcare is a relatively recent consideration, in comparison to the substantial existing literature emphasizing the importance of senior leadership [62]. Nevertheless, a growing body of evidence shows that middle management’s position in the organization centres their contribution, particularly their ability to facilitate communication throughout the organization [63, 64].

The communication ‘role’ has been posited as an area where the middle manager influences change in organizations. An important example of this would be the work of Birken et al., who highlight the significance of middle managers in implementing evidence-based innovations [63, 65]. Though Birken’s model also considers non-communication aspects (such as mediating between strategy and operational activities for employees), the model centres on the importance of diffusing, synthesizing, and ‘selling’ information related to innovation implementation [63, 65].

Additional literature finds that middle managers leverage their access to knowledge and networks to serve as a ‘conduit’, transferring information regarding the strategy down through the lower levels of the organization [63], while simultaneously translating the strategy or vision into actionable processes and steps to adopt by front-line employees [66–68]. Middle managers are also able to direct the flow of information both upward and downward (per Floyd & Wooldridge’s model [68], discussed further below), communicating from the front lines back up to senior leadership.

## Toward a conceptual/theoretical understanding of middle management’s role in change initiatives in healthcare

Kotter’s model of change [69] presents a useful framework for understanding how middle management can leverage their position in a changing environment. Though middle managers can play an important role in almost every step of the model, communication is specifically cited in the first step, in creating a sense of urgency in the organization. In an analysis of Kotter’s model, Appelbaum et al. elaborate on the communication paradigm by explaining that, among other factors, frequency of communication about the change in the organization is required to increase urgency about the change [70]. This again aligns with the evidence supporting the middle manager’s role as the ‘conduit’ between senior management and front line employees in the organization. If the middle manager has proper knowledge, abilities, and resources from senior management, they will be able to generate the necessary frequency of communication regarding the upcoming change. The 4th stage of Kotter’s model also references communication, whereby management must communicate the change vision to reduce uncertainty – or as stated by Appelbaum et al. [70] “tell people, in every possible way and at every opportunity, about the why, what and how of the changes” (p.766). The middle management role is again indirectly emphasized, highlighting that two-way communication is far more effective than one-way, honing in on a strength of this specific level of management [69].

In order for middle managers to successfully leverage these knowledge and communication skills, they must both understand their role and possess the requisite ability to leverage their position in the organization [71]. This is emphasized through the middle managers’ understanding of the role itself, via role conflict and ambiguity [72]. ‘Role theory’, then, suggests that understanding of their role is a determinant of middle managers’ ability to effectively implement strategy within their organization. This theory is based on the works of Floyd & Wooldridge [12, 73, 74] who presented a seminal framework of middle management roles. In brief, this model outlines the bidirectional role of middle management in strategy implementation, whereby they can influence upward (senior leadership) or downward (immediate or indirect subordinates) and through different communication strategies [68].

Although this framework is useful in understanding the inherent ambiguity of the middle manager’s role as well as the potential ways to leverage this role, it does not explain how or why middle managers choose to leverage their influence (i.e., whether they enact more convergent or divergent communication strategies). Another theory that may be useful to explicate this phenomenon is social capital theory [75–77]. Very broadly, *organizational* social capital “consists of the *structural* (connections among actors), *relational* (trust between actors), and *cognitive* (shared goals and values among actors) dimensions of the relationships between organizational members” [78] (P.584, emphasis from authors). These structural, relational, and cognitive factors directly impact middle managers’ ability to strategically manoeuvre within the organization, where structures either facilitate or hinder the middle manager’s positioning [79]. Embedded within the social capital theory is the concept of social networks [80], which emphasizes the strength of connections within and between organizations. Depending on the strength and breadth (both vertically and laterally) of the middle manager’s network within the organization, their ability to access knowledge and communicate (or transfer) it effectively may be facilitated or constrained. Through social capital theory, it may be understood that middle managers are more or less effective based on the strength of their social networks and their organizational social capital. However, it could also be suggested that the strength of each network may influence which elements of the typology are adopted by the middle manager (e.g., based on their perceived ability to influence upwards or downwards more effectively).

Although social capital theory presents an important framework for considering the individual role and potential efficacy of middle managers, it is not sufficient in providing a macro- or organization-level view of middle managers’ in facilitating radical change.

As is evident from our discussion above, much of the literature on middle management’s contribution to organizational change emphasizes its *position* in the organization. Indeed, all of the aforementioned skills and capabilities required of middle managers to influence change leverage their position as intermediaries with access to knowledge and insights from both the executive and front-line levels of the organization. In light of this, we propose a theoretical framework exploring middle management’s contribution to organizational culture change that not only examines this group’s skills and capabilities, but also ‘situates’ these capabilities in the wider context of the organization, thus offering a means to understand when and how middle managers will operate most effectively. We suggest that organizational learning theory and the ‘exploration versus exploitation’ paradigm offers a meaningful avenue to explain this context, particularly within the frame of patient safety.

## Organizational learning theory as a framework for middle management

Organizational learning theory is uniquely suited to address the various contextual factors associated with the middle manager’s role [26]. Organizational learning theory is a meta-theory that considers the context in which learning about new knowledge takes place, and describes learning as the outcome of social processes. It suggests that individual-level factors, macro-environmental factors, and the nature of the knowledge itself influence how learners perceive action-outcome (cause-effect) relationships [81–83]. Organizational learning theory is highly relevant to understanding knowledge translation phenomena [84–89]. Some learning can be observed as changes in worker behaviours and work processes, while other learning cannot be observed since it leads to decisions to maintain the status quo. Learning about new knowledge - that in turn begets new knowledge - in organizations can endure well beyond the tenure of individual workers who contribute to it: it can be embedded in formal policies and procedures, role descriptions, and information systems [82, 90, 91]. It can also be captured in less explicit forms including informal communication channels, culture, and behavioural norms [85, 92].

Organizational learning has garnered a great deal of research attention since it is related to organizational performance [81, 93]. Performance improvements in organizations are realized with accrued experience and enables organizations to knowingly learn and adapt their work routines [94]. This ability to “adaptively learn” is observed in health care settings [95]. Adaptive learning can accrue over time with no overt effort, or it can be intentional. Some organizations intentionally acquire new knowledge (e.g., evidence-based practice guidelines) from outside and create leaning opportunities (e.g., in the form of a pilot test) with the expectation that the ensuing learning will be adaptive – or beneficial to the organization. The performance variation that exists in every industry [82, 96] is suggested to be attributable, at least in part, to differences in organizations’ capacity to learn [82], related to how they learn [97] and the resources that they make available for learning.

### Exploration vs. exploitation

In organizational learning theory, organizations can be ‘exploration’ focused, ‘exploitation’ focused, or ‘ambidextrous’ where they create some combination of both strategies. Exploration refers to organizations’ activities of searching, innovating, and risk-taking, while exploitation refers to efficiency, selection, and implementation [26]. Each of these in turn affects the organizational structures that the firm selects and develops, as a result of the overarching learning strategy it chooses to pursue, with the end goal of creating a competitive advantage based on their chosen lens.

Since its inception, this exploration/exploitation paradigm has been associated with an implicit dichotomy: how can an organization avoid the trade-offs of too much of either exploration or exploitation? Organizations that focus solely on exploration at the expense of exploitation are “likely to find that they suffer the costs of experimentation without gaining many of its benefits. They exhibit too many undeveloped new ideas... Conversely, systems that engage in exploitation to the exclusion of exploration are likely to find themselves trapped in suboptimal stable equilibria” [26] (P.71). This inherent dichotomy or structural tension is intuitively reminiscent of the tension observed in healthcare organizations and in the health services literature [98, 99], whereby leaders and providers are admonished to innovate while at the same time sustaining an acceptable level of patient safety. To emphasize this idea, one study presented a proposed model of the structures and ideologies that would encompass either an exploration- or exploitation-based organization [100]. In essence, this model demonstrates how significantly an organization is impacted by adopting an exploration or exploitation focus – from levels of hierarchy and centralization in the organization through modes of communication, even impacting at the micro level of group processes, determining whether groups will be goal-oriented (exploitation) or adaptable (exploration) [100].

In an attempt to address this issue, numerous authors have presented varying models of the ‘ambidextrous organization’, which successfully incorporates elements of both exploration and exploitation [29, 101, 102]. Though many iterations exist of how both elements would function in a singular organization, the dominant paradigm is that of Benner & Tushman [29], whereby both elements can exist within sub-units of the organization, generally with very limited integration between these sub-units and the larger organizational type [103].

### Middle managers as ambidextrous actors in organizations

In terms of organizational change, exploration has been associated with ‘radical’ innovation, while exploitation has been linked only to incremental change [103]. In this vein, Posen and Levinthal [104] presented a model of exploration and exploitation specific to dynamic environments or turbulent environmental change. Though the model is highly comprehensive and a full overview is beyond this paper, the important focus is that change is a moving target, rather than an end goal [104]. This notion ties directly into efforts at changing patient safety cultures: initial change efforts may require all-encompassing initiatives, however these efforts will never officially ‘end’, in that healthcare organizations will always have to uphold their standard of patient safety, facilitated by a strong PSC. In light of this, organizations need to adopt strategies that allow them to ‘chase the moving target’ without dedicating all of their resources to repeated change and quality improvement initiatives. This paper proposes that the adoption of a successful ambidextrous organizational structure – in particular for a healthcare organization – is best accomplished not through division of units, rather through division of ‘levels’ in the organization, capitalizing on the importance of middle management. Since middle managers are uniquely able to acquire and communicate knowledge across the organization, they are able to impact both strategic and operational outcomes.

Based on the theory of organizational learning and the concept of exploration vs. exploitation, a series of propositions are presented as they relate to the middle manager’s role in creating an ambidextrous healthcare organization to implement radical organizational change. The mechanics of this role are explained by the following propositions, whereby mid-level managers are situated between senior leadership that is strategy- or exploration-focused, and front-line workers that are operations- or exploitation-focused.

#### Propositions

As mentioned above, the Institute of Medicine’s reports were fundamental in changing the nature of acute healthcare organizations as well as their basic understanding of what constitutes proper, patient-focused and safe care [30, 31]. It could be posited that since then, acute healthcare organizations have adopted a perpetual exploration focus in their efforts at improvement and have become stuck in a cycle of ‘perpetual pilots’ [105]; the explosion of quality improvement initiatives and programs aimed at fostering patient safety and patient-centered care support the assumption that organizations have shifted their strategies to reflect these new goals. It could further be proposed that the end result of this focus has become exactly as originally predicted: organizations are suffering the costs of exploration (unfinished, unsustained, and costly ideas [106–108]) without reaping the benefits of long-term change. Furthermore, Posen and Levinthal emphasized the diminishing returns of constantly remaining in a state of exploration, where only so many changes can take place in an organization within a relatively short timespan [104]. This context for acute care organizations further emphasizes the expectation for senior leadership to be entirely exploration-focused when undergoing radical organizational change. Additionally, if the existing climate in healthcare is focused on innovation (notably in terms of technological innovation), it follows that policies and funding would support this structural change. As a result, senior leadership would be expected to dedicate all resources towards the focus on explorative organizational activities. Therefore, the first proposition is:

##### Proposition 1: Senior leadership in acute healthcare organizations engage primarily in exploration in their strategic activities

Given this organizational context, this then allows middle managers to adopt a different strategic focus than the remainder of the organization. Middle managers are not required to be as focused on vision and strategic planning as senior leaders, but still have the knowledge and context to focus on strategic priorities more so than would front-line employees. Given that their role most often involves bringing high-level ideas into actionable tasks for employees, middle managers may very well have a different focus than the rest of the organization when it comes to influencing organizational change. That is, middle managers serve an important role in balancing out the exploration vs. exploitation dynamic in a changing organization. As senior management is constantly focusing on vision, strategic planning, or any number of new changes occurring in the health system, it is the middle managers who a) translate the vision and strategy into action, and b) juggle the balancing act between the innovation focus of senior leadership and the operational efficiency required to function in a day-to-day environment. These roles assure that middle managers themselves adopt an ambidextrous view of the organization, engaging in both the explorative activities of senior leadership and the exploitative patient-facing activities of front-line workers. Not only does this leverage the skill and position of middle management, but it also assures that senior leadership is able to focus on developing truly radical notions of change in PSC, that are based on the reality of on-the-ground experiences of front-line workers.

##### Proposition 2: Healthcare organizations will create stronger patient safety cultures when middle management adopts an ambidextrous view of change and innovation strategic activities

Elaborating on the above proposition, there are two issues to consider: first, per this theoretical framework, there needs to be a different focus at each ‘level’ of the organization in order to function optimally and assure that both sides of the change management paradigm (radical vs. incremental change) are being considered. Second, if senior management specifically isn’t focused on exploration, it will be near impossible for middle management to adopt change or innovation, particularly with no high-level representative to champion the change; the only exception to this might be if the middle managers were part of specific sub-units tasked with radical change, per Benner and Tushman’s proposition [103].

##### Proposition 2a: Proposition 2 will only hold true when senior leadership is focused on exploration

As mentioned earlier, part of the proposition of the ambidextrous healthcare organization is not only that senior leadership is exploration-focused, but also that front-line workers are exploitation-focused. This delineation between exploration-focused senior leadership and exploitation-focused front-line workers is uniquely critical in the healthcare field, as organizations can be seen as struggling to achieve a balance between the need for radical innovation and equally resounding calls to improve patient safety. When considering these competing priorities from the perspective of the front-line worker, sustaining patient safety efforts requires an emphasis on implementing best practices and clinical guidelines, which leaves little time to focus on activities aimed at influencing organizational change (as demonstrated by quality improvement literature, which identifies lack of time as a barrier to front-line staff implementing quality improvement activities [109]). Given the nature of healthcare providers’ already over-burdened schedules and difficulty keeping updated on the influx of such clinical guidelines [110], this only further hinders the possibility of embedding innovations and exploration-seeking activities into their practices.

##### Proposition 3: Front-line healthcare workers will generally adopt an exploitation-focus in performing their day-to-day and patient-facing activities

The different focus at each level of the organization, we suggest, creates a dynamic ‘tension’ in the organization (based on the organization’s fundamental activities and structures) that – similar to the flow of communication – exists in a bidirectional relationship. That is, when each level is focused on a different goal, a continuous feedback loop between the two can only reinforce the change process, which is ultimately the proposed role of the middle manager. Using the example of PSC, if middle management is able to effectively implement processes that improve patient outcomes, they can then communicate this to senior leadership allowing for real-time evaluation of the culture change effort and giving leadership the leverage to seek out ‘bolder’ change strategies that complement the demonstrated success of the original change. All this, without sacrificing the benefits of sustainable improvement.

##### Proposition 3a: The greater the ‘tension’ between exploration and exploitation at different levels of an organization, the more successful the organization will likely be in implementing patient safety culture change

ᅟ

### Relating exploration and exploitation to antecedents of middle management’s influence

Our propositions reinforce the idea that exploration/exploitation can clarify the context in which middle management functions most effectively. That is, when middle managers understand their strategic role in the organization, ambiguity and confusion regarding their role would likely be significantly reduced. As well, this framework lends itself well to the typology of middle management laid out by Floyd & Wooldridge [68], whereby the ambidextrous focus can explain what types of communications will take place both upstream and downstream, and how effective they will be in facilitating strategy implementation.

### Limitations and avenues for future research

The primary limitation of our work is that it presents a model largely based outside of the healthcare literature, and as such its applicability is not assured. However, our review of the literature demonstrating factors associated with PSC change support the theoretical effectiveness of this model, particularly the important role of leadership at multiple organizational levels. As well, certain studies have emphasized the unique position of the middle manager in communicating vision and ‘trickling down’ organizational change goals. This evidence supports the exploration/exploitation framework in predicting middle managers’ most effective behaviours in influencing innovation implementation, and in particular the innovation of PSC.

Regarding the role of the exploration/exploitation model of middle management, to be sure this model cannot account for all antecedents of middle management’s effectiveness, notably when considering individual antecedents. Nevertheless, the model does frame the variety of contextual factors in a meaningful way to better understand how middle managers will be able to successfully implement organizational change.

Finally, this theoretical framework draws on a number of assumptions that need to be tested empirically, not the least of which includes that healthcare organizations have in fact adopted a primarily exploration-based focus. This should be addressed in future research, as well as the assumption of the present model that middle managers will be most effective when they adopt an ambidextrous focus. Future research in this area may not only involve defining which lens is optimal for each level of the organization, but also looking at how the interconnections and the aforementioned tensions at all three levels work together, perhaps as a necessary antecedent to change

## Conclusion

This paper presents a new model to understand the influential role of middle management in influencing organizational change, specifically in the context of patient safety culture. The extant literature on leadership at the mid-level of organizations, both within and beyond healthcare was used to frame a series of propositions for middle management’s contribution to radical organizational change. Through this, we suggest a new framework based on organizational learning theory and the exploration vs. exploitation paradigm; this framework suggests that, through an ambidextrous approach to organizational change strategy, middle managers can leverage their unique position in the organization to most effectively influence patient safety change. This organizational learning perspective offers a novel way of framing the mid-level managers’ role, through both explorative and exploitative activities, that contributes to our knowledge of the role of middle managers in organizational change.

## Abbreviations

KM: Knowledge Management
PSC: Patient Safety Culture
